# Supplementary material for: Prevalence of dyslipidemia and associated risk factors among adult residents of Mekelle City, Northern Ethiopia
Source: PLoS One. 2021 Feb 9;16(2):e0243103. doi: 10.1371/journal.pone.0243103 (PMC7872241; doi:10.1371/journal.pone.0243103)
Supplement: Attachment — Submitted filename: Response to Reviewers.docx [file pone.0243103.s002.docx]

**Response to Reviewers**

| **Journal requirements** | **Reviewers’ and academic editor’s comments** | **Response** | **Page and Line number**  **(on Manuscript)** | **Page and Line number**  **(on Track Changes)** |
| --- | --- | --- | --- | --- |
| 1 | Please ensure that your manuscript meets PLOS ONE's style requirements. | Manuscript prepared according to PLOS ONE's style | The whole document | The whole document |
| 2 | Your ethics statement must appear in the Methods section of your manuscript. | Ethics statement incorporated in the Methods and materials section. | Page 5  Line 93-99 | Page 6  Line 124-130 |
| **Reviewer 1** | | | | |
| 1 | Given the definition of the prevalence of the disease in a community, it cannot be said that this study examined the prevalence of dyslipidemia in Ethiopia. | We admit the comment and addressed as follows.  This study was aimed to estimate the prevalence of dyslipidemia and its associated risk factors among adults residing in Mekelle city, Northern Ethiopia. | Page 2/Line 24-25 and  Page 4/Line 79-81 | Page 2/Line 28-30 and  Page 5/Line 101-103 |
| 2 | What was the inclusion criteria? | Being an adult aged 20 years and above and residents who lived at least 6 months in the city were considered as the inclusion criteria. | Page 5  Line number 89-90 | Page 6  Line number 118-120 |
| 3 | From line 219 to line 241 the text is added because most of the explanations are in Table 1 and there is no need for explanation in the text. More appropriate and concise sentences can be used instead. For example, most people were married and the marital status of men and women was significantly different (P = 0.001), women consumed significantly more vegetables during the week than men (P = 0.007). | Addressed as recommended.  We have indicated only the most important findings from table 1 in the text. | Page 10  Line number 196-210 | Page 14-15  Line number 278-308 |
| 4 | In line 204, the name of the test is Hosmer, not Hesmer | Addressed as recommended.  The goodness of fit of the model was checked using the Hosmer-Lemeshow test at p>0.05. | Page 9  Line number 181 | Page 12  Line number 252 |
| 5 | Many parts of an article have a very long text. | We make the article short and precise without missing the most important content. | The whole document | The whole document |
| **Reviewer 2** | | | | |
| 1 | Carefully check for spelling errors and proper grammatical usage throughout the manuscript and correct. Lines 124-128 read somewhat disjointed and are confusing to the reader (possible footnotes for further description?). Please clarify this information. | We made extensive revision for spelling and grammatical errors and modified accordingly.  The information in Lines 124-128 is clarified as recommended. | page 6  Line number 118-120 | Page 8  Line number 156-158 |
| 2 | Under Methods: Move ' Ethical Issues' subsection information into the 'Study Design and Participants' subsection at the beginning of the Methods section. | ‘Ethical issues’ moved to the recommended subsection (under study design, setting, and participants) | Page 5  Line number 93-99 | Page 6  Line number 124-130 |
| 3 | Considering that roughly ~10% of participants could not read/write, how was consent given from these participants? Was proxy or next-of-kin consent given for these participants? Were they read the consent form specifically? Mention how the study gained consent in this subpopulation and list this important detail in the Methods. | Data collectors read the information to each illiterate participant and asked him or her for voluntary participation. If voluntary, they put their fingerprint as a signature in the written consent form. This is the usual practice in developing countries, where many people are illiterate.  This information is indicated in the Ethical issues subsection under materials and methods. | Page 5  Line number 96-97 | Page 6  Line number 127-129 |
| 4 | While table 3 does a great job addressing the covariable relationship between several characteristics of the participants and dyslipidemia, an interesting comparison should be made to include "Alcohol use" which was significantly greater in the cohort. Because alcohol can dramatically alter lipid metabolism, this comparison should be made. | We have included alcohol use in Table 3 and Table 5 according to reviewer’s recommendation. However, the effect is not statistically significant as expected. The possible reason might be the frequency, type, and amount of alcohol consumed matters. Since alcohol is expensive, most people in the study area consumed few numbers of bottle of beer per week, which may not have significant effect on the lipid profile of individuals. | Page 15  Line number 279-280  Page 19  Line number 345-346 | Page 21-22  Line number 408-409  Page 27-28  Line number 516-517 |
| 5 | TABLE 1: The far left column is confusing to read. Recommend a headline for the far left column as "Characteristics" and show each characteristic in the subcolumn either outlined with separate borders or place break lines between characteristics. As written, the wording seems to run together and is difficult to distinguish transitions to the next characteristic listed. | Addressed as recommended.  Two separate columns have been created (named as ‘variables’ and ‘categories’). | Page 11  Line number 216-217 | Page 15-16  Line number 318-319 |
| 6 | TABLE 3: Add borders around the p-value row. As mentioned above, it is difficult to identify and read the table as presented. Consider alternative ways to organize the data, or at the very least, add more break lines and subcolumns to organize the presentation in a clearer manner. | Table 3 has been re-organized as recommended. | Page 15  Line number 279-280 | Page 21-22  Line number 408-409 |
| 7 | Discussion: Line 453: Discuss how characteristics related to household income (e.g. House ownership, wealth, house servant, laundry machine) are related to the prevalence of dyslipidemia. Also, significant alcohol use in the cohort is a limitation that should be addressed. | Having better household assets is related with better access to energy dense foods, physical inactivity, engagement in low intensity work, low energy expenditure all of which may lead to weight gain and accumulation of excessive fat, which in turn may lead to dyslipidemia.  More than one-quarters of participants in the present study were alcohol consumers and we tried to assess the association with dyslipidemia according to your recommendation. However, the effect is not significant as expected. The possible reason might be the frequency, type, and amount of alcohol consumed matters. Since alcohol is expensive, most people consume few numbers of bottle of beer per week, which may not have significant effect on the lipid profile of individuals. | Page 23  Line number 419-428 | Page 32  Line number 607-616 |
