# Supplementary material for: Prevalence of dyslipidemia and associated risk factors among adult residents of Mekelle City, Northern Ethiopia
Source: PLoS One. 2021 Feb 9;16(2):e0243103. doi: 10.1371/journal.pone.0243103 (PMC7872241; doi:10.1371/journal.pone.0243103)
Supplement: Attachment — Submitted filename: Response to Reviewers.docx [file pone.0243103.s003.docx]

**Response letter**

**Dear Editor:**

I have sent the revised manuscript entitled **“Prevalence of dyslipidemia and associated risk factors among adult residents of Mekelle City, Northern Ethiopia.”** I really appreciate for your critical review process. All the raised comments are very helpful in improving the manuscript. We have addressed all the comments raised by an editor and the reviewer in the manuscript and we have summarized in the following table. Besides, we tried to shorten the whole manuscript and major revision has been made on grammar and syntax.

| **S.N** | **Reviewers’ and academic editor’s comments** | **Response** | **Page and Line number**  **(on the Manuscript without track change)** | **Page and Line number**  **(on the manuscript with track change)** |
| --- | --- | --- | --- | --- |
| 1 | It is best to add a reference to line 102, To determine what is the formula for calculating the sample size. | 12. Lwanga S, Lemeshow S. Sample size determination in health studies: a practical manual. England: World Health Organization; 1991. | Page 5  Line 102 | Page 6  Line 119 |
| 2 | The “Materials and methods” section is very long and should be summarized. For example,   - if possible,” Study design and setting” And “Study population”, it is better to combine them and write more briefly. Also, - if possible,” Selection and training sections of data collectors” And “Data collection and quality control”, it is better to combine them and write more briefly. Also, - if possible,” Blood sample collection and laboratory analysis” And “Definition of terms”, it is better to combine them and write more briefly. | We have revised the Materials methods section. We have combined and briefly wrote the sub-sections based on your recommendation. As much as possible we make it short and precise. | Page 4-5  Line 84-90 | Page 5  Line 99-106 |
|  |  |  | Page 6-7  Line 116-137 | Page 7-8  Line 137-168 |
|  |  |  | Page 7-8  Line 140-158 | Page 8-10  Line 172-200 |
| 3 | Please shorten the text wherever possible in all parts of the manuscript. For example in line 172, the phrase “Sample characteristics were expressed as mean ± standard deviation (SD) for continuous variables, and frequencies and percentages for categorical variables” can be omitted because this is specified in the results section. | The indicated phrase is deleted from the data analysis part. Besides, the whole manuscript is revised to minimize the content. Unnecessary repetitions have been deleted. | Page 8  Line 161-171 | Page 10  Line 203-217 |
| 4 | In “Result” section, As mentioned in the previous review, items that are clear from the tables should not be repeated in the text. Please provide brief explanations in the text only in cases where the difference is significant. It is better to remove the phrase “the mean + SD age of the participants was 38.99 + 14.22 years” in line 197. It is better to remove the phrase “More than half (54.8%) of participants were women.” in line 197. | Unnecessary phrases are deleted from all table descriptions based on your recommendation. Explanation is provided only for variables with significant difference throughout the manuscript. | Page 9  Line 179-185 | Page 11-12  Line 236-255 |
| 5 | In line 199 to 203, additional text has been written about marital status, occupation, smoking and alcohol, and a brief explanation can be written instead.  For example, please briefly explain that men and women were significantly different in terms of marriage, occupation, smoking and alcohol (P<0.005).  And only give a brief explanation if you see an interesting result for each of them. Please correct lines 203 to 210 as described above. | We have addressed the comment as you have recommended. | Page 9  Line 179-180 | Page 12  Line 240-244 |
|  |  |  | Page 9  Line 179-185 | Page 11-12  Line 236-255 |
| 6 | In line 207, in the table of this frequency, 13.1 is written, but in the text, 13.4. | Thank you for your critical observation. We have corrected it (13.1%). | Page 9  Line 183 | Page 12  Line 251-252 |
| 7 | In line 221, the phrase "slightly higher among men (68.3%) compared to women (65.3%)" It is better to remove it because it is not more common in men. | Removed as recommended. | Page 10  Line 192-197 | Page 14  Line 286-304 |
| 8 | Please delete these phrases because they are clear from the table: Line 222 “Half (49.5%) of the study participants had elevated LDL-C while 30.8% had an elevated total cholesterol level” Line 226 “More than one fourth (27.1%) of the participants were overweight.” Line 230 “Almost one-thirds (30.5%) and 18.7% of the participants were hypertensive and pre-hypertensive respectively” Line 233 “The mean + SD BMI and WC were 24.4+4.9 kg/m2 and 85.6+13.6 cm respectively, with no significant difference across gender” Line 259 “The prevalence of dyslipidemia was 57.8%, 79.3%, and 95.2% among normal, overweight, and obese participants respectively” Also is in lines 260 to 264. | All phrases indicated in your comment have been deleted based on your recommendation. We really appreciate your critical review. | Page 10  Line 192-197 | Page 14  Line 286-304 |
| 9 | In line 229, it is better to write that the difference between men and women was significant. | Addressed | Page 10  Line 193-195 | Page 14  Line 289-291 |
| 10 | In line 257 It cannot be said that the behavior of TG and HDL with the age variable, is as mentioned. | Though the difference in TG was significant with age, there was inconsistency. Whereas, HDL-C was not significantly different with age.  Addressed as recommended. | Page 11  Line 215-216 | Page 16-17  Line 325-326 |
| 11 | In line 264, this phrase is the type of phrase at the beginning of the paragraph. It is better to combine them and bring them in one sentence: "The risk of dyslipidemia and its component lipid abnormalities were also consistently increased with FBG and blood pressure." | We have combined the two sentences as you have commented. | Page 11  Line 212-214 | Page 16  Line 314-323 |
| 12 | In Table 2, there is a discrepancy in the findings! For the “Waist to hip ratio” in qualitative state, it is observed that a higher percentage of women have high values of Waist to hip ratio. But in quantitative state, we see that the average of this variable is higher in men. It’s not logical. | This is due to the difference in the cutoff point. Men have greater cutoff (0.9) than women (0.8). The same is true for waist circumference, with a cutoff 94 cm and 80 cm for men and women, respectively. Besides, women have naturally greater hip circumference relative to their waist circumference due to excessive fat accumulation around the hip compared to men, which makes women to have lower cutoff. Therefore, significantly **higher proportion of women** have raised waist to hip ratio (≥0.8) and men have significantly **higher mean value** of waist to hip ratio. | Page 10  Line 193-195 | Page 14  Line 289-299 |

Sincerely yours.

Gebremedhin Gebreegziabiher (corresponding author)

Email: [ghingherg@gmail.com](mailto:ghingherg@gmail.com),

Mobile: +251914754562,

Nov-03/2020
